# Extracellular vesicles derived from bone marrow mesenchymal stem cells loaded on magnetic nanoparticles delay the progression of diabetic osteoporosis via delivery of miR-150-5p

**DOI:** 10.1007/s10565-022-09744-y

**Published:** 2022-09-16

**Authors:** Chen Xu, Zhaodong Wang, Yajun Liu, Bangguo Wei, Xiangyu Liu, Keyou Duan, Pinghui Zhou, Zhao Xie, Min Wu, Jianzhong Guan

**Affiliations:** 1 Department of Orthopedics, the First Affiliated Hospital of Bengbu Medical College, No. 287, Changhuai Road, Bengbu, 233000 Anhui Province People’s Republic of China; 2 Anhui Province Key Laboratory of Tissue Transplantation (Bengbu Medical College), Bengbu, 233000 Anhui Province People’s Republic of China; 3 Jinan University, Guangzhou, 510000 Guangdong Province People’s Republic of China; 4 Third Military Medical University of Chinese PLA, Chongqing, 400038 People’s Republic of China

**Keywords:** Diabetic osteoporosis, Bone marrow mesenchymal stem cells, Magnetic nanoparticles, Extracellular vesicles, MicroRNA-150-5p, MMP14, Wnt/β-catenin pathway, Osteogenesis

## Abstract

Extracellular vesicles derived from bone marrow mesenchymal stem cells (BMSC-EVs) are emerged as carriers of therapeutic targets against bone disorders, yet its isolation and purification are limited with recent techniques. Magnetic nanoparticles (MNPs) can load EVs with a unique targeted drug delivery system. We constructed gold-coated magnetic nanoparticles (GMNPs) by decorating the surface of the Fe_3_O_4_@SiO_2_ core and a silica shell with poly(ethylene glycol) (PEG)-aldehyde (CHO) and examined the role of BMSC-EVs loaded on GMNPs in diabetic osteoporosis (DO). The osteoporosis-related differentially expressed miR-150-5p was singled out by microarray analysis. DO models were then established in Sprague–Dawley rats by streptozotocin injection, where poor expression of miR-150-5p was validated in the bone tissues. Next, GMNP_E_ was prepared by combining GMNPs with anti-CD63, after which osteoblasts were co-cultured with the GMNP_E_-BMSC-EVs. The re-expression of miR-150-5p facilitated osteogenesis in osteoblasts. GMNP_E_ could promote the enrichment of EVs in the bone tissues of DO rats. BMSC-EVs delivered miR-150-5p to osteoblasts, where miR-150-5p targeted MMP14 and consequently activated Wnt/β-catenin pathway. This effect contributed to the enhancement of osteoblast proliferation and maturation. Furthermore, GMNP_E_ enhanced the EV-based delivery of miR-150-5p to regulate the MMP14/Wnt/β-catenin axis, resulting in promotion of osteogenesis. Overall, our findings suggest the potential of GMNP-BMSC-EVs to strengthen osteoblast proliferation and maturation in DO, showing promise as an appealing drug delivery strategy against DO.

**Graphical abstract:**

1. GMNPs-BMSCs-EVs-miR-150-5p promotes the osteogenesis of DO rats.

2. miR-150-5p induces osteoblast proliferation and maturation by targeting MMP14.

3. Inhibition of MMP14 activates Wnt/β-catenin and increases osteogenesis.

4. miR-150-5p activates the Wnt/β-catenin pathway by downregulating MMP14.

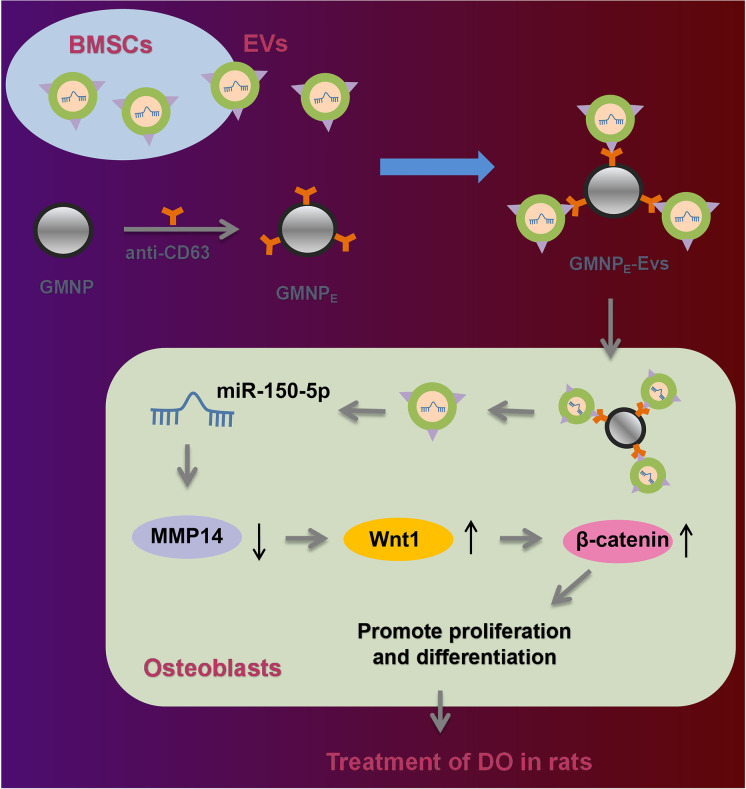

**Supplementary Information:**

The online version contains supplementary material available at 10.1007/s10565-022-09744-y.

## Introduction

Diabetes mellitus often impairs the skeletal system (Schacter and Leslie 2017). The risk of developing bone disorders, including osteoporosis, is thus greatly increased (Cortet et al. 2019; Rehling et al. 2019). Available biological agents to treat diabetic osteoporosis (DO) consist of anti-diabetic drugs, anti-osteoporosis drugs, and anti-resorptive osteoporosis drugs (Mohsin et al. 2019). The advent of new and original molecular biology techniques has shed a light on the novel molecular mechanisms that underlie the development of individual treatment plans.

Extracellular vesicles (EVs) derived from mesenchymal stem (stromal) cells (MSCs) (MSC-EVs) can deliver therapeutic targets for various diseases, showing a promise as an alternative cell-free therapy (Rani et al. 2015). Despite this, the isolation and detection of EVs still exhibit several technical drawbacks such as limited sensitivity and time consumption (Oliveira-Rodriguez et al. 2017). Magnetic nanoparticles (MNPs) with surface decorated with stimuli-responsive polymers can enable efficient and rapid EV separation (Jauregui et al. 2018). MNPs are a class of nanomaterials, showing diverse applications, such as magnetic separation, magnetic imaging, magnetic biosensing (diagnostics), and drug and gene delivery as they (Wu et al. 2019), highlighting a promise of MNP-loaded EVs for disease treatment.

MicroRNAs (miRNAs) exhibit therapeutic use by controlling gene expression of target mRNAs (Liu et al. 2021a). It should be noted that EVs contain miRNAs, which have been reported to be essential for the mediation of intercellular communication and as potential therapeutic candidates for disease (Mori et al. 2019). miR-150 can trigger the osteoblastic phenotype related to osteoblast function and bone mineralization, which shows therapeutic potential of promoting bone formation in some diseases, especially in osteoporosis (Dong et al. 2015). miR-150-5p has been identified to target matrix metalloproteinase 14 (MMP14) and downregulated its expression in hepatoma cells (Li et al. 2014). Pharmacologic inhibition of MMP14 can reduce bone resorption and potentiate bone gain (Delgado-Calle et al. 2018). Activation of the human MMP14 gene promoter links to enhanced nuclear translocation of β-catenin (Lu et al. 2014). Of note, activation of the Wnt/β-catenin pathways in human bone marrow MSCs (BMSCs) results in promotion of osteogenesis (Pang et al. 2022). In addition, activation of this pathway can promote osteoblastic differentiation, thus preventing the development of osteoporosis (Sharma and Nam 2019; Yang et al. 2020).

In light of these described above, MSC-EVs containing miR-150-5p may play a critical role in the progression of DO via the MMP14/Wnt/β-catenin axis. In this study, we utilized nanoparticles consisted of Fe_3_O_4_, SiO_2_, poly(ethylene glycol) (PEG), and aldehyde (CHO) (GMNPs) to load the EVs isolated from BMSCs and carried out co-culture assays to address this hypothesis.

## Methods

### Establishment of rat models of DO

Seventy male SD rats (7–8 weeks old; 180 ± 20 g) were housed with 60–70% relative humidity at 25 ± 2 °C under a 12-h light/dark cycle. They were free access to food and water. Following 7 days of acclimatization, 6 rats were injected with normal saline and used as control and 64 rats were randomly selected for DO modeling by intraperitoneal injection with streptozotocin (STZ; 60 mg/kg, Sigma-Aldrich, St Louis, MO) (Wang-Fischer and Garyantes 2018; Cao et al. 2021; An et al. 2019). The study, approved by the Animal Ethics Committee of the First Affiliated Hospital of Bengbu Medical College (approval no.: 2021–245), was conducted according to the Guide for the Care and Use of Laboratory Animals published by the US National Institutes of Health.

After 72 h, random blood glucose > 16.7 mmol/L was indicative of successful modeling. Bone mineral density (BMD) and bone mineral content (BMC) were assessed every 4 weeks using dual-energy X-ray absorptiometry (DXA; Hologic, Marlborough, MA). The significant statistical difference in BMD and BMC between the two groups denoted successful establishment of DO (Supplementary Fig. 1).

After 8 weeks, 55 rats were successfully modeled, among which, 6 rats were randomly selected and treated with GMNP_E_-EVs (*n* = 3) or EVs (*n* = 3); the remaining 48 rats were treated with lentiviral vector harboring mimic negative control (NC), inhibitor NC, miR-150-5p mimic, or miR-150-5p inhibitor, PBS alone, or co-treated with GMNP_E_-EV-mimic NC + lentiviral vector harboring sh-NC, GMNP_E_-EV-miR-150-5p mimic + lentiviral vector harboring shRNA (sh)-NC, and GMNP_E_-EV-miR-150-5p mimic + lentiviral vector harboring sh-β-catenin (*n* = 6 for rats upon each treatment). GMNP_E_ (10 mg/kg body weight in 400 μL PBS), EVs (100 μL), and lentiviral vector (100 μL) were injected into rats via tail vein, and N52 neodymium magnet was then used to provide external magnetic field on rat femur, with the flowing GMNP_E_ collected to the femur. Lentivirus was purchased from Sangon Biotechnology Co., Ltd. (Shanghai, China). After 8 weeks, femoral tissues were collected for subsequent micro-CT and other tests.

### Isolation and culture of primary BMSCs and osteoblasts

BMSCs were isolated as previously described (Huang et al. 2021). The bilateral tibia and femur of rats were collected, and the bone marrow was washed with DMEM/F12 to obtain a mixed cell suspension. The suspension was subsequently centrifuged (1000 r/min, 5 min) and a precipitate containing BMSCs was harvested, then re-suspended in DMEM/F12 and cultured with 5% CO_2_ at 37 °C. For the characterization of BMSCs (Duan et al. 2020; Dominici et al. 2006), alizarin red S staining was performed to test the calcium deposition at the 3rd week of osteogenic differentiation induction, so as to examine the osteogenesis of BMSCs, and oil red O staining was applied to observe the lipid droplets at the 3rd week of adipogenic differentiation induction to assess the adipogenesis of BMSCs. Cell morphology and growth were observed and identified under an inverted microscope (Supplementary Fig. 2).

The osteoblasts were isolated from trabecular bone fragments by sequential enzymatic digestion (Huang et al. 2021; Zhao et al. 2010). The morphology of cells was finally observed with an inverted microscope (Supplementary Fig. 3).

### Cell transfection

Upon attaining about 75% confluence, cells were transfected with miR-150-5p mimic, mimic NC, miR-150-5p inhibitor, inhibitor NC, oe-NC, oe-MMP14, sh-NC, sh-MMP14#1, sh-MMP14#2, sh-MMP14#3, sh-β-catenin#1, sh-β-catenin#2, and sh-β-catenin#3 alone or in combination (Supplementary Table 1).

The gene overexpression plasmid pCMV6-AC-GFP was purchased from Shanghai Yaji Biotechnology Co., Ltd. (YC-13849RJ), and the shRNAs from Thermo Fisher Scientific.

### RT-qPCR

Total RNA was extracted with TRIzol method and reverse-transcribed into cDNA with PolyA Tailing Reverse Transcription Kit (B532451, Sangon; for miRNA detection) or RevertAid RT Reverse Transcription Kit (K1691, Thermo Fisher Scientific; for mRNA detection). RT-qPCR was followed using Applied Biosystems QuantStudio System (4,489,084, Thermo Fisher Scientific). As normalized to U6 or GAPDH, the fold changes were calculated using the 2^−ΔΔCt^ method (Livak and Schmittgen 2001) (Supplementary Table 2).

### Immunoblotting

Total protein was extracted, separated, and transferred onto PVDF membranes. The membrane was blocked using 5% skimmed milk powder and underwent incubation with primary antibodies to CD63 (1:1000, rabbit, PA5-92,370, Thermo Fisher Scientific), HSP70 (1:1000, mouse, ab2787, Abcam), TSG101 (1:1000, rabbit, ab125011, Abcam), GM130 (1:500, rabbit, PA5-95,727, Thermo Fisher Scientific), MMP14 (1:1000, rabbit, MA5-32,076, Thermo Fisher Scientific), β-catenin (1:5000, rabbit, ab32572, Abcam), Wnt1 (1:1000, rabbit, PA5-85,217, Thermo Fisher Scientific), RUNX2 (mouse, ab76956, Abcam), BSP (1:1000, rabbit, PA5-114,915, Thermo Fisher Scientific), OPN (1 µg/mL, rabbit, ab63856, Abcam), OCN (1–10 µg/mL, mouse, MA1-20,786, Thermo Fisher Scientific), and β-actin (1:5000, mouse, ab8226, Abcam). The membrane was re-probed with HRP-labeled secondary antibody IgG (goat anti-rabbit, ab205718, 1:20,000, Abcam) or goat anti-mouse (ab6789, 1:5000, Abcam). Following ECL development, quantification was conducted with the ImageJ 1.48 software, with β-actin as the normalization.

### ELISA

Using rat ELISA kit of tartrate-resistant acid phosphatase (TRAP) (BS-E11304R2, Boshen Biotechnology Co., Ltd., Jiangsu, China) and rat ELISA kit of C-terminal telopeptide of type I collagen (CTX-I) (XY-SJH-DS1027, Xuanya Biotechnology Co., Ltd., Shanghai, China), we detected levels of CTX-I and TRAP5b in the serum of rats.

### Isolation and identification of EVs from BMSCs

The EVs were isolated from BMSCs by differential ultracentrifugation-based method as previously described (Liao et al. 2019).

The obtained EVs were then observed under a TEM (Hitachi H-7650, BAHENS, Shanghai, China) (Zhou et al. 2018). Nanoparticle Tracking Analyzer ZetaView_Particle Metrix (DKSH, China) was adopted to detect size distribution of EVs (Kooijmans et al. 2013). Furthermore, immunoblotting was conducted to examine the EV surface marker proteins (CD63, HSP70, TSG101, and GM130).

### Co-culture of BMSC-EVs with osteoblasts and uptake of EVs by osteoblasts

BMSCs were transiently transfected with Cy3-labeled miR-150-5p, and then co-cultured with osteoblasts using a Transwell co-culture system (Guan et al. 2020; Zhu et al. 2019). After nucleus staining with Hoechst 33,342, uptake of the labeled EVs by osteoblasts was visualized by a confocal laser scanning microscope (ZEISS LSM 800).

### Detection of osteoblast viability, mineralized nodules, and ALP activity

For viability assay of osteoblasts, CCK-8 kit (10 μL, C0038, Beyotime, Shanghai, China) was applied to assess cell viability (Ge et al. 2019). Osteoblasts were fixed and then stained with 1% alizarin red or ALP staining solution to observe the number of mineralized nodules or ALP activity respectively (Li et al. 2021).

### Synthesis and characterization of nanoparticles

Various chemical substances were fixed on the surface of MNPs (102,138, XFNANO, Nanjing, Jiangsu, China) to produce GMNPs or RhB-labeled GMNPs (Supplementary Table 3). The Fe_3_O_4_@SiO_2_-PEG-CHO (GMNPs or RhB-labeled GMNPs) was obtained according to previous report (Liu et al. 2020).

The intermediate products and final products were analyzed by SEM (S-4800, Hitachi, Shanghai Fulai Optical Technology Co., Ltd., Shanghai, China) with energy-dispersive spectrometer (IQLAAHGABMFAAWMACL, Thermo Fisher Scientific) and Fourier transform infrared spectrometer (912A0770, Thermo Fisher Scientific). DLS (Nano ZS90, Malvern) was applied for size and size distribution measurement.

### Preparation and biocompatibility of GMNPE

GMNP_E_ was generated with GMNPs (Fe_3_O_4_@SiO_2_-PEG-CHO) bound by the rabbit antibody to CD63 (PA5-92,370, Thermo Fisher Scientific). In short, 1 mg/mL GMNP solution was purified by magnetic separation, and incubated with anti-CD63 antibody overnight at 4 °C to obtain GMNP_E_. Fluorescent GMNP_E_ was prepared using the same method, with the aforementioned GMNPs replaced by RhB-labeled GMNPs.

In order to assess drug carrying capacity of GMNP_E_, thermogravimetric analysis (TGA; STA 6000, PerkinElmer) was conducted for analyzing MNPs, GMNPs, and GMNP_E_. The adsorption capacity and stability of HSA were measured with FITC-labeled HSA by fluorescence spectroscopy.

### In vitro identification of GMNPE-EV binding and targeting efficiency

Purified EVs were labeled with PKH67 (green) (MINI67, Sigma). After magnetic separation, GMNP_E_-EVs were obtained, with the morphology observed by a high-resolution TEM (200 kV, CM200, Philips). Furthermore, RhB and PKH67 were separately used to label GMNP_E_ and EVs to confirm this binding (Liu et al. 2020).

Immunoblotting was applied for confirmation of magnetic separation of serum EVs. Samples were probed with CD63 (rabbit, PA5-92,370, Thermo Fisher Scientific) and then with IgG (goat anti-rabbit, ab205718, 1:20,000, Abcam).

### Immunofluorescence

GMNP_BSA_ and GMNP_E_ were fixed in 4% paraformaldehyde, blocked, and probed with rabbit antibody to CD63 (PA5-92,370, Thermo Fisher Scientific). The cells were re-probed with goat anti-rabbit (#60,839, 1:50, Cell Signaling Technologies, Beverly, MA) conjugated to Alexa Fluor® 555. Finally, images were captured under an 80i fluorescence microscope.

### Uptake of GMNPE-EVs by osteoblasts

GMNP_E_-EVs (RhB-labeled GMNP_E_, PKH67-labeled EVs) were incubated with osteoblasts for 3 h at 37 °C and fixed with PFA. Cytoskeleton staining was performed to observe the localization of GMNP_E_-EVs in the cells, and the nuclei were counterstained with Hoechst 33,342.

### Flow cytometry

FITC-labeled GMNP_E_ or PKH67-labeled GMNP_E_-EVs containing different ratios of anti-CD63 were co-cultured with osteoblasts in PBS at 4 °C for 30 min. These samples were analyzed using a FC500 flow cytometer (Morey Biosciences, Inc., Shanghai, China). The FlowJo v.10 (Tree Star) software was applied for data processing.

### Dual-luciferase reporter assay

3′-UTR sequence of MMP14 with predicted miR-150-5p binding sites was cloned into pGL3-basic vector (6107, Jiran Biotechnology Co., Ltd. Shanghai, China) to generate luciferase reporter vector pGL3-basic MMP14-3′-UTR-WT and pGL3-basic MMP14-3′-UTR-MUT. The reporter vectors underwent co-transfection with mimic NC or miR-150-5p mimic into HEK-293 T cells (Biobw, Beijing, China). Luciferase activity, as normalized to renilla luciferase, was determined using Dual-Luciferase Reporter Assay System (Shenzhen TOP Biotechnology Co., Ltd., Shenzhen, Guangdong, China).

### Immunofluorescence detection of GMNPE-EV uptake in rat femoral tissues

GMNP_E_-EVs (RhB-labeled GMNP_E_, PKH67-labeled EVs) were injected into DO rats via tail vein. An N52 neodymium magnet was used to provide an external magnetic field in the rat femur, and the flowing GMNP_E_ nanoparticles were collected to the femur. Next, the rats were euthanized and the femoral, brain, liver, and kidney tissues were removed to prepare longitudinal frozen sections, which were subjected to immunofluorescence staining with OCN (10 µg/mL, mouse antibody, 33–5400, Thermo Fisher Scientific) and 80i fluorescence microscopy.

### Immunohistochemistry and hematoxylin–eosin staining

The femoral specimens were decalcified with 18% EDTA and cut into 5-μm-thick sections. The sections were immunostained with OCN (10 µg/mL, mouse, 33–5400, Thermo Fisher Scientific) and type I collagen (1:100, rabbit, 600–406-103, Thermo Fisher Scientific), and observed with a microscope (Olympus CX31, TUSEM, Shanghai, China). HE staining was used for histological observation as previously described (Liu et al. 2021b).

### Histomorphometric analysis

On days 10 and 3 before euthanasia, the rats were intraperitoneally injected with 0.1% calcein (10 mg/kg body weight, C0875, Sigma-Aldrich) dissolved in PBS. After euthanasia, the femurs were removed from the rats. The samples were fixed, dehydrated, embedded in methyl methacrylate, and cut into 60-μm-thick sections. The double labeling of calcein was observed under a fluorescence microscope and the Image-Pro Plus 6 software was applied to measure mineral apposition rate (MAR) of trabecular bones.

### Micro-CT scanning

Femoral tissues were scanned with mCT-40 micro-CT system (Scanco Medical, Switzerland) to analyze femoral tissue growth (Zuo et al. 2019). Image reconstruction was performed by the NRecon software (Bruker, Kontich, Belgium), and data were analyzed using the CTAn program (Bruker). The following parameters were determined: trabecular thickness (Tb.Th), bone volume/tissue volume (BV/TV), trabecular number (Tb.N), and trabecular separation (Tb.Sp).

### Statistical analysis

Unpaired *t*-test, one-way ANOVA and two-way ANOVA, or repeated measures ANOVA with Tukey’s tests were utilized to calculate statistical significance for two-group, multi-group, and time-based data. All results processed using the SPSS 21.0 software were presented as mean ± SD. *p* < 0.05 suggests statistically significant difference.

## Results

### miR-150-5p promotes osteogenesis in DO rats

**Fig. 1:**
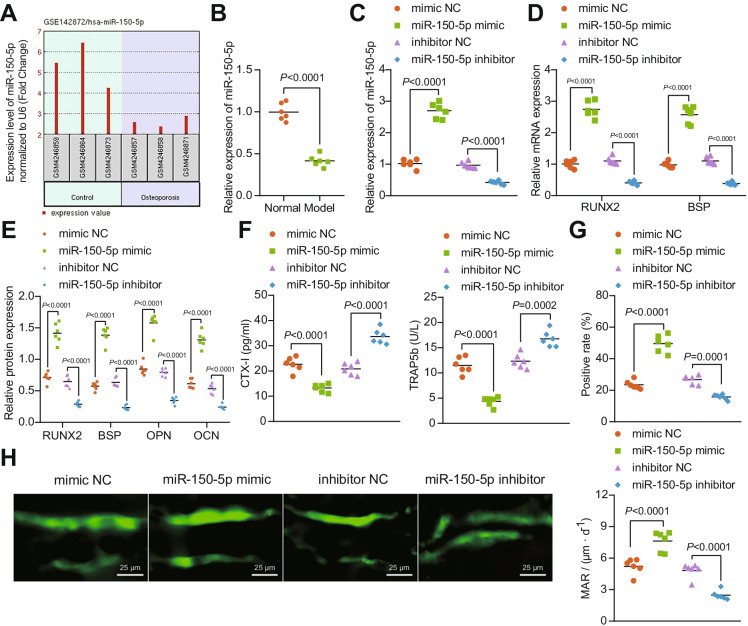
The promoting effect of miR-150-5p on the osteogenesis in DO rats. **A** Differential expression of miR-150-5p in the GSE142872 dataset (3 serum samples from osteoporosis patients and 3 serum samples from healthy individuals) from GEO database (https://www.ncbi.nlm.nih.gov/gds). Differential analysis was conducted using R language “limma” package with *p* < 0.05 as the threshold to identify differentially expressed genes. **B** miR-150-5p expression determined by RT-qPCR in the bone tissues of normal and DO rats. **C** miR-150-5p expression determined by RT-qPCR in the bone tissues of miR-150-5p mimic or miR-150-5p inhibitor-treated DO rats. **D** mRNA expression of RUNX2 and BSP determined by RT-qPCR in the bone tissues of miR-150-5p mimic or miR-150-5p inhibitor-treated DO rats. **E** Immunoblotting of RUNX2, BSP, OPN, and OCN proteins in the bone tissues of miR-150-5p mimic or miR-150-5p inhibitor-treated DO rats. **F** Serum levels of CTX-I and TRAP5b in miR-150-5p mimic or miR-150-5p inhibitor-treated DO rats determined by ELISA. **G** Immunohistochemistry analysis of the rate of positive osteoblasts in miR-150-5p mimic or miR-150-5p inhibitor-treated DO rats. **H** Immunofluorescence staining analysis (calcein double labeling) for the bone mineralization rate on the trabecular bone surface in miR-150-5p mimic or miR-150-5p inhibitor-treated DO rats. * *p* < 0.05. *n* = 6

Analysis of the GSE142872 dataset from GEO database identified the significantly poorly expressed miR-150-5p in the serum samples of patients with osteoporosis (Fig. 1A). Consistently, lower expression of miR-150-5p was witnessed in the bone tissues of DO rats than normal rats (58%; Fig. 1B). Moreover, miR-150-5p mimic successfully elevated the expression of miR-150-5p in DO rats (164%), while miR-150-5p inhibitor led to a notable reduction (57%; Fig. 1C).

The osteogenic-specific markers RUNX2 and BSP were increased to 172% and 162%, respectively at mRNA levels in response to miR-150-5p overexpression but downregulated to 63% and 65% respectively in its inhibitor presence (Fig. 1D). Immunoblots indicated that forced expression of miR-150-5p elevated the levels of osteogenesis-related proteins RUNX2, BSP, OPN, and OCN, while a reversed effect was induced by miR-150-5p inhibition (Fig. 1E, Supplementary Fig. 4A).

Meanwhile, the serum levels of CTX-I and TRAP5b were reduced to 41% and 62% respectively upon miR-150-5p overexpression, and miR-150-5p inhibition resulted in elevations of CTX-I (61%) and TRAP5b (36%) (Fig. 1F). Based on immunohistochemistry and immunofluorescence, forced expression of miR-150-5p led to increases in the rate of OCN-positive osteoblasts (111%) and bone mineralization rate on the trabecular bone surface (46%), while declines (OCN to 41% and bone mineralization to 49%) were noted following miR-150-5p loss-of-function (Fig. 1G, H).

The above-said results indicate that miR-150-5p restores the osteogenesis of DO rats.

### BMSC-EV-miR-150-5p facilitates the proliferation and maturation of osteoblasts

**Fig. 2:**
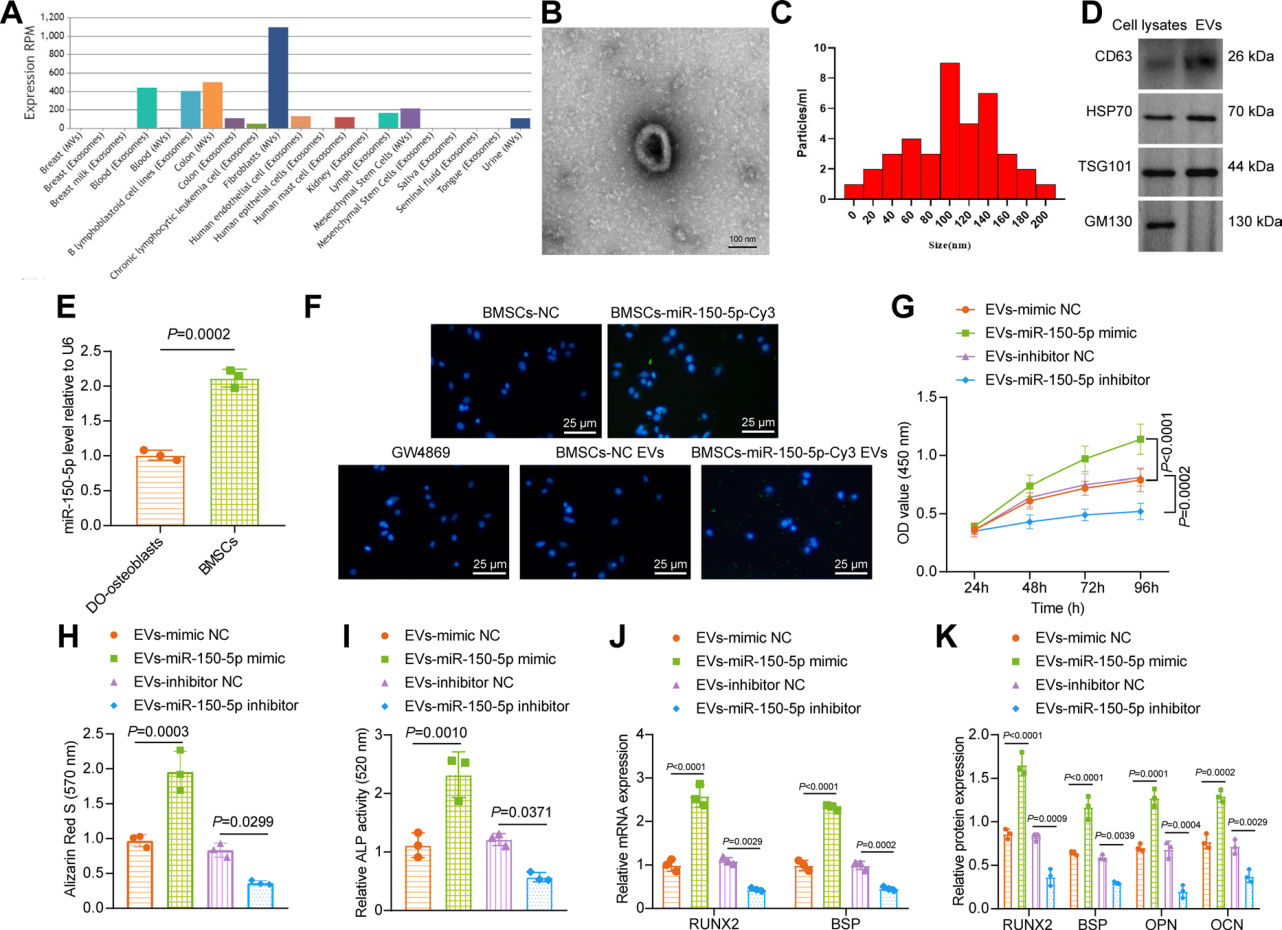
BMSC-EV-miR-150-5p stimulates the proliferation and maturation of osteoblasts. **A** Expression of miR-150-5p in MSC-EVs predicted by the EVmiRNA website (http://bioinfo.life.hust.edu.cn/EVmiRNA#!/). **B** Morphological characterization of the isolated EVs observed using a TEM. **C** The size distribution of the isolated EVs analyzed by nanoparticle tracking analysis. **D** Immunoblotting of EV surface makers CD63, HSP70, and TSG101 along with negative EV marker GM130 in the isolated EVs. **E** miR-150-5p expression in osteoblasts isolated from DO rats and BMSCs determined by RT-qPCR. **F** Cy3-labeled green fluorescence signal observed under a fluorescence microscope. **G** Osteoblast viability of osteoblasts co-cultured with EV-miR-150-5p mimic or EV-miR-150-5p inhibitor measured by CCK-8 assay. **H** Alizarin red S staining of mineralized nodules in osteoblasts co-cultured with EV-miR-150-5p mimic or EV-miR-150-5p inhibitor. **I** ALP staining of ALP activity in osteoblasts co-cultured with EV-miR-150-5p mimic or EV-miR-150-5p inhibitor. **J** mRNA expression of RUNX2 and BSP in osteoblasts co-cultured with EV-miR-150-5p mimic or EV-miR-150-5p inhibitor determined by RT-qPCR. **K** Immunoblotting of RUNX2, BSP, OPN, and OCN proteins in osteoblasts co-cultured with EV-miR-150-5p mimic or EV-miR-150-5p inhibitor. * *p* < 0.05. The cell experiment was repeated 3 times independently

The EVmiRNA website predicted that miR-150-5p was enriched in MSC-EVs (Fig. 2A). Under a TEM, the isolated vesicles sourced from BMSCs exhibited a representative double-layer membrane structure, cup-shaped or flat-shaped, with diameters of about 100 nm (Fig. 2B, C). Immunoblotting results showed expression of CD63, HSP70, and TSG101 in EVs but the absence of GM130 in the isolated vesicles (Fig. 2D), demonstrating the typical characteristics of EVs. In addition, expression of miR-150-5p was notably higher in BMSCs compared with osteoblasts isolated from DO rats (Fig. 2E).

The BMSCs transiently transfected with Cy3-labeled miR-150-5p mimic were co-cultured with osteoblasts. After 72 h, the osteoblasts showed obvious Cy3 fluorescence, which became disappeared in response to the addition of EV release inhibitor GW4869. Green fluorescence was also detected in the osteoblasts co-cultured with EVs from the Cy3-labeled miR-150-5p mimic-transfected BMSCs (Fig. 2F). Accordingly, miR-150-5p could be transmitted into osteoblasts through BMSC-EVs.

Furthermore, we observed enhanced viability (44%), as well as mineralized nodules and ALP activity (1.1 folds) of osteoblasts co-cultured with EV-miR-150-5p mimic, while a decline (36%) was observed after co-culture with EV-miR-150-5p inhibitor caused diminished viability (36%), mineralized nodules, and ALP activity (0.5 folds) (Fig. 2G-I, Supplementary Fig. 5A, B).

Additionally, EV-mediated delivery of miR-150-5p in osteoblasts exhibited elevated mRNA expression of RUNX2 and BSP, along with enhanced protein levels of RUNX2, BSP, OPN, and OCN, and the delivered miR-150-5p inhibitor undermined the effects (Fig. 2J-K, Supplementary Fig. 4B).

Altogether, BMSC-EVs deliver miR-150-5p to heighten the proliferation and maturation of osteoblasts.

### Synthesis and characterization of GMNPs

SEM images showed increased size of spherical Fe_3_O_4_ and Fe_3_O_4_@SiO_2_ (MNPs) with rough surface changed into relatively smooth after PEG modification at 200 nm (Supplementary Fig. 6A). The energy-dispersive spectrum analysis of GMNPs showed the existence of Fe_3_O_4_, SiO_2_, PEG, and hydrazine bond in GMNPs (Supplementary Fig. 6B). Infrared spectroscopy revealed the expected characteristic peaks of the functional groups during each synthesis step, including the modification of Fe_3_O_4_ by SiO_2_, hydrazone, and PEG-CHO (Supplementary Fig. 6C).

Zeta potential measurement data presented that the surface charge gradually changed according to the electronic properties of varied functional groups (Supplementary Fig. 6D). The magnetic measurement results showed that these nanoparticles were superparamagnetic without evident hysteresis loop before and after modification (Supplementary Fig. 6E), indicating that the effect of surface modification on the core structure could be negligible. X-ray diffraction pattern results also supported this finding (Supplementary Fig. 6F). Compared with anti-CD63 antibody that directly bound to MNPs without PEG modification, namely Fe_*3*_O_*4*_@SiO_*2*_@anti-CD63 antibody (MNP_E_), the stability of GMNP_E_ was improved (Supplementary Fig. 6G). Hence, the aforesaid results indicate the successful synthesis of GMNPs.

### GMNPE promotes the enrichment of EVs in the bone tissues of DO rats

We combined the anti-CD63 antibody targeting EVs to aldehyde-functionalized magnetic nanoparticles to form a Schiff base between the aldehyde group and the primary amine of the antibody, and finally negatively charged GMNP_E_ nanoparticles were formed. Hence, negative charge of GMNP_E_ is conducive to repelling the negatively charged proteins during blood circulation. Immunofluorescence results showed the presence of anti-CD63 antibody on the surface of GMNP_E_ (Supplementary Fig. 7A). In addition, TGA showed antibodies accounting for 54% of GMNP_E_ (Supplementary Fig. 7B).

GMNP_E_ were subsequently co-cultured with EVs. TEM observation results showed an obvious core–shell-corona structure of GMNP_E_-EVs, accompanied by obvious membrane EVs with vesicle-like structure (Supplementary Fig. 7C). Fluorescence confocal microscopic observation further confirmed the ability of GMNP_E_ (RhB-labeled GMNP_E_ and PKH67-labeled EVs) to capture EVs, wherein EV-related fluorescence (green) and GMNP_E_-related fluorescence (red) were co-localized (Supplementary Fig. 7D).

Next, the EV binding capacity of GMNPs containing anti-CD63 was assessed when substituted with IgG isotype control antibody (GMNP_N_) as a control. Compared with GMNP_N_, the capture of EVs by GMNP_E_ was significantly enhanced (Supplementary Fig. 7E).

In addition, immunoblotting results showed the presence of CD63 in GMNP_E_-EV samples, which was same as the positive control, whereas CD63 was absent in GMNP_N_-EV samples (Supplementary Fig. 7F). These results confirmed that GMNP_E_ could capture EVs. Next, EVs or GMNP_E_-EVs were co-cultured with osteoblasts, and the results suggested that GMNP_E_ considerably raised the number of EVs in osteoblasts (Supplementary Fig. 7G). Furthermore, confocal microscopic images showed that injection with GMNP_E_-EVs led to more EVs enriched in the osteoblasts of femoral tissues of DO rats than injection with EVs alone (Supplementary Fig. 7H). In the other tissues such as brain, heart, lung, liver, spleen, and kidney, GMNP_E_-EV signal was barely detected (Supplementary Fig. 7I).

Therefore, GMNP_E_ may potentiate the enrichment of EVs in the bone tissues of DO rats.

### GMNPE-EV-miR-150-5p enhances proliferation and maturation of osteoblasts

**Fig. 3:**
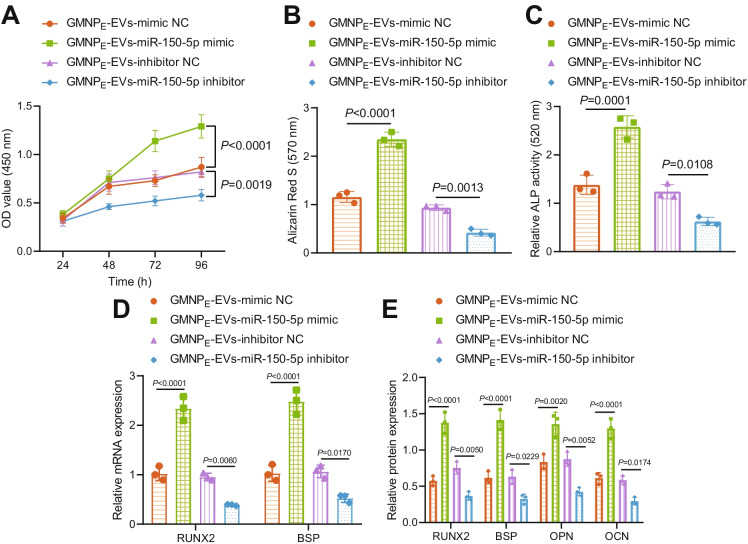
GMNP_E_-EV-miR-150-5p enhances the proliferation and maturation of osteoblasts. Osteoblasts were co-cultured with GMNP_E_-EV-miR-150-5p mimic or GMNP_E_-EV-miR-150-5p inhibitor. **A** Osteoblast viability measured by CCK-8 assay. **B** Alizarin red S staining of mineralized nodules in the osteoblasts. **C** ALP staining of ALP activity in the osteoblasts. **D** mRNA expression of RUNX2 and BSP in the osteoblasts determined by RT-qPCR. **E** Immunoblotting of RUNX2, BSP, OPN, and OCN proteins in the osteoblasts. * *p* < 0.05. The cell experiment was repeated 3 times independently

We next aimed to characterize the role of GMNP_E_-EV-miR-150-5p in the biological functions of osteoblasts. The viability (48%), mineralized nodules, and ALP activity (87%) of osteoblasts were enhanced upon GMNP_E_-EV-miR-150-5p mimic, while notable declines were witnessed in viability (29%), mineralized nodules, and ALP activity (50%) upon GMNP_E_-EV-miR-150-5p inhibitor (Fig. 3A-C, Supplementary Fig. 5C, D).

Additionally, mRNA expression of RUNX2 and BSP and the protein levels of RUNX2, BSP, OPN, and OCN in osteoblasts were raised by GMNP_E_-EV-miR-150-5p mimic, and GMNP_E_-EV-miR-150-5p inhibitor reversed the effects (Fig. 3D-E, Supplementary Fig. 4C).

The aforementioned findings highlighted the pro-proliferative and pro-mature effects of GMNP_E_-EV-miR-150-5p on osteoblasts.

### miR-150-5p stimulates the proliferation and maturation of osteoblasts by targeting MMP14

**Fig. 4:**
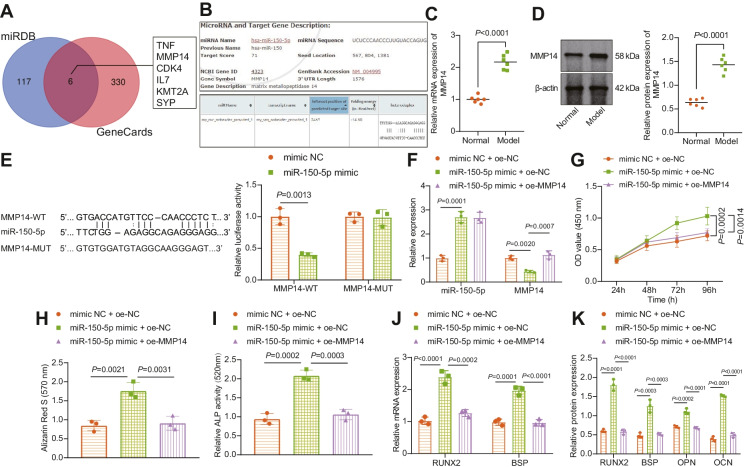
miR-150-5p strengthens the proliferation and maturation of osteoblasts by binding to MMP14. **A** Venn plot (http://bioinformatics.psb.ugent.be/webtools/Venn/) of downstream genes of miR-150-5p predicted by the miRBD website (http://mirdb.org/) and the osteogenesis-related genes predicted by the GeneCards website (relevance score ≥ 20; https://www.genecards.org/). **B** Binding sites between MMP14 and hsa-miR-150-5p as well as rno-miR-150-5p. **C** MMP14 mRNA expression in the bone tissues of normal and DO rats determined by RT-qPCR. **D** Immunoblotting of MMP14 protein in the bone tissues of normal and DO rats. *n* = 6*.* **E** Binding of miR-150-5p to MMP14 verified by dual-luciferase reporter assay in HEK-293 T cells. **F** miR-150-5p expression and MMP14 mRNA expression in osteoblasts in the presence of with miR-150-5p mimic alone or combined with oe-MMP14 determined by RT-qPCR. **G** Osteoblast viability in the presence of with miR-150-5p mimic alone or combined with oe-MMP14 measured by CCK-8 assay. **H** Alizarin red S staining of mineralized nodules in osteoblasts in the presence of with miR-150-5p mimic alone or combined with oe-MMP14. **I** ALP staining of ALP activity in osteoblasts. **J** mRNA expression of RUNX2 and BSP in osteoblasts in the presence of with miR-150-5p mimic alone or combined with oe-MMP14 determined by RT-qPCR. **K** Immunoblotting of RUNX2, BSP, OPN, and OCN proteins in osteoblasts in the presence of with miR-150-5p mimic alone or combined with oe-MMP14. * *p* < 0.05. The cell experiment was repeated 3 times independently

We then intended to determine the mechanism of miR-150-5p in osteoblasts. miRBD website showed 123 downstream genes of miR-150-5p, which were then submitted to Venn diagram analysis to attain the intersection genes with the osteogenesis-related genes predicted by the GeneCards database. Six genes were found at the intersection, namely, TNF, MMP14, CDK4, IL7, KMT2A, and SYP (Fig. 4A). More importantly, binding sites were observed between MMP14 and hsa-miR-150-5p as well as rno-miR-150-5p (Fig. 4B). Therefore, we selected MMP14 as the target for the subsequent experiments.

In addition, higher expression of MMP14 was observed at the mRNA and protein in the bone tissues of DO rats versus normal rats (Fig. 4C, D). Luciferase analysis revealed a decline in the luciferase activity of MMP14-WT but that of MMP14-MUT was not affected following miR-150-5p mimic (Fig. 4E).

Moreover, MMP14 mRNA expression was decreased in cells overexpressing miR-150-5p, which was rescued by additional treatment with oe-MMP14 (Fig. 4F). Consistently, the miR-150-5p overexpression-induced increases in viability (43%), mineralized nodules, and ALP activity (121%) were reversed by restoration of MMP14 (viability by 25%, mineralized nodules, and ALP activity by 49%) (Fig. 4G-I, Supplementary Fig. 5E, F).

Additionally, miR-150-5p overexpression-induced elevation in the mRNA expression of RUNX2 and BSP along with protein expression of RUNX2, BSP, OPN, and OCN were also reversed by re-expression of MMP14 (Fig. 4J, K, Supplementary Fig. 4D).

Overall, miR-150-5p targets MMP14 and inhibits its expression, augmenting the proliferation and maturation of osteoblasts.

### Inhibition of MMP14 activates Wnt/β-catenin and promotes proliferation and maturation of osteoblasts

**Fig. 5:**
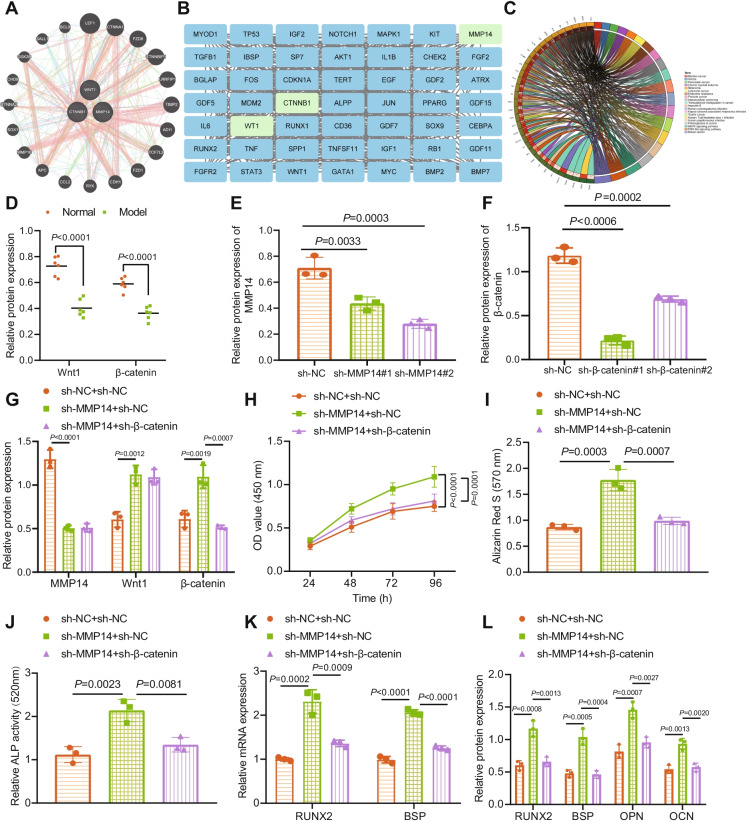
Inhibition of MMP14 facilitates proliferation and maturation of osteoblasts by activating the Wnt/β-catenin pathway. **A** PPI among MMP14, Wnt1, and CTNNB1 predicted by GeneMANIA website (http://genemania.org/). **B** PPI network of MMP14, Wnt1, CTNNB1, and osteogenesis-related genes constructed by STRING database (https://string-db.org/) with the Cytoscape 3.6.0 software (https://cytoscape.org/) applied for visualization. **C** KEGG enrichment analysis of the candidate genes using R “ClusterProfiler” package. **D** Immunoblotting of Wnt1 and β-catenin proteins in the bone tissues of normal and DO rats. *n* = 6*.* **E** Immunoblotting to testify the silencing efficiency of sh-MMP14 in osteoblasts. **F** Immunoblotting to testify the silencing efficiency of sh-β-catenin in osteoblasts. **G** Immunoblotting of MMP14, Wnt1, and β-catenin proteins in osteoblasts in the presence of sh-MMP14 alone or combined with sh-β-catenin. **H** Osteoblast viability in the presence of sh-MMP14 alone or combined with sh-β-catenin assessed by CCK-8 assay. **I** Alizarin red S staining of mineralized nodules in osteoblasts in the presence of sh-MMP14 alone or combined with sh-β-catenin. **J** ALP staining of ALP activity in osteoblasts in the presence of sh-MMP14 alone or combined with sh-β-catenin. **K** mRNA expression of RUNX2 and BSP in osteoblasts in the presence of sh-MMP14 alone or combined with sh-β-catenin determined by RT-qPCR. **L** Immunoblotting of RUNX2, BSP, OPN, and OCN proteins in osteoblasts in the presence of sh-MMP14 alone or combined with sh-β-catenin. * *p* < 0.05. The cell experiment was repeated 3 times independently

Next, we proceeded to elucidate the molecular mechanism of MMP14 involved in osteogenesis. The GeneMANIA website predicted interaction between MMP14, Wnt1, and CTNNB1 (Fig. 5A). At the same time, the top 50 genes related to osteogenesis were screened by the GeneCards website. A protein–protein interaction (PPI) network of MMP14, Wnt1, and CTNNB1 and osteogenesis-related genes was constructed by STRING database, where MMP14, Wnt1, and CTNNB1 were found to be closely related to osteogenesis (Fig. 5B). KEGG results showed that MMP14 can mediate the Wnt/β-catenin pathway (Fig. 5C). However, it is unknown whether this MMP14-mediated pathway functioned in the osteogenesis process.

Immunoblotting results demonstrated lower protein levels of Wnt1 (45%) and β-catenin (38%), the core proteins of the Wnt/β-catenin pathway, in the bone tissues of DO rats than normal rats (Fig. 5D, Supplementary Fig. 4E). Additionally, immunoblotting confirmed the silencing efficiency of shRNAs targeting MMP14 and β-catenin in osteoblasts as shown by decreased expression of MMP14 and β-catenin, of which sh-MMP14#2 and sh-β-catenin#1 showed the superior silencing efficiency (MMP14 expression decreased to 82% and β-catenin expression to 42%) (Fig. 5E, F, Supplementary Fig. 4F, G) and were utilized for subsequent experiments.

Silencing of MMP14 by sh-MMP14 led to elevated Wnt1 and β-catenin protein levels. Additional sh-β-catenin treatment caused no changes in the MMP14 and Wnt1 protein expression while downregulating β-catenin protein expression (Fig. 5G, Supplementary Fig. 4H).

The MMP14 knockdown-induced increases in viability (50%), mineralized nodules, and ALP activity (91%) of osteoblasts were counterweighed by simultaneous knockdown of β-catenin by 26% in viability and by 37% mineralized nodules and ALP activity (Fig. 5H-J, Supplementary Fig. 5G, H).

Additionally, β-catenin silencing could reverse the enhancements in RUNX2 and BSP mRNA expression and RUNX2, BSP, OPN, and OCN protein expression induced by MMP14 silencing (Fig. 5K, L, Supplementary Fig. 4I).

Together, suppression of MMP14 may contribute to Wnt/β-catenin activation, thus augmenting the proliferation and maturation of osteoblasts.

### GMNPE-EV-miR-150-5p promotes the osteogenesis in DO rats through the MMP14/Wnt/β-catenin axis

**Fig. 6:**
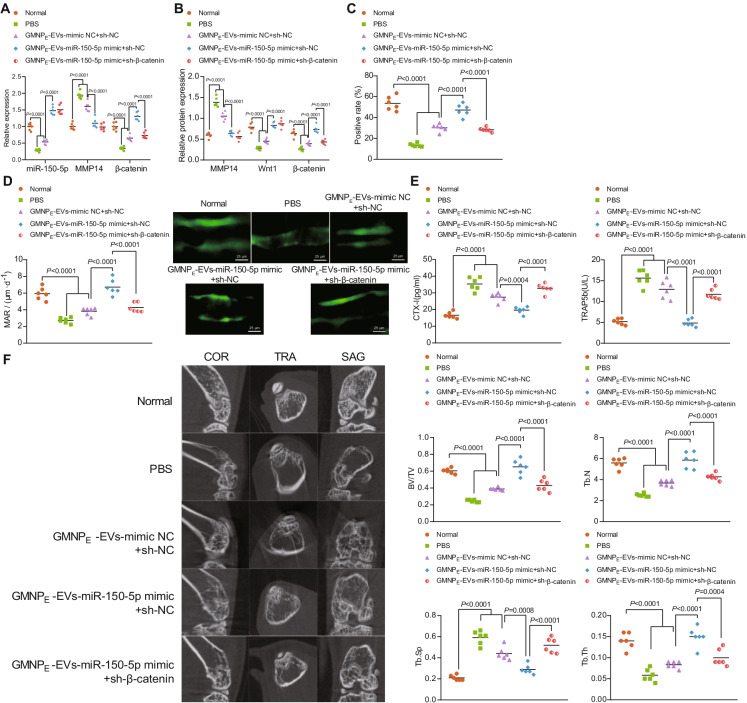
GMNP_E_-EV-miR-150-5p enhances the osteogenesis of DO rats through the MMP14/Wnt/β-catenin axis. The DO rats were treated with GMNP_E_-EV-miR-150-5p mimic alone or combined with sh-β-catenin. **A** miR-150-5p expression and mRNA expression of MMP14 and β-catenin in the bone tissues of DO rats determined by RT-qPCR. **B** Immunoblotting of MMP14, Wnt1, and β-catenin proteins in the bone tissues of DO rats. **C** Immunohistochemistry analysis for the number of osteoblasts. **D** Immunofluorescence staining analysis (calcein double labeling) for the bone mineralization rate on the trabecular bone surface. **E** Serum levels of CTX-I and TRAP5b in DO rats determined by ELISA. **F** Micro-CT analysis of the BV/TV, Tb.Th, Tb.N, and Tb.Sp in the bone tissues (coronal section, sagittal section, and transverse section) of DO rats. * *p* < 0.05. *n* = 6

Finally, we intended to characterize the effect of GMNP_E_-EV-miR-150-5p on the osteogenesis in DO rats through the MMP14/Wnt/β-catenin axis. Of note, lower levels of miR-150-5p and β-catenin yet higher MMP14 level were observed in the bone tissues of DO rats treated with either PBS or GMNP_E_-EV-mimic NC + sh-NC than normal rats. miR-150-5p and β-catenin expression was elevated while MMP14 expression was diminished in the bone tissues of DO rats upon treatment with GMNP_E_-EV-miR-150-5p mimic. Conversely, the elevation in β-catenin levels by GMNP_E_-EV-miR-150-5p mimic was offset by sh-β-catenin (Fig. 6A).

In addition, consistent protein levels were obtained as shown by immunoblotting results (Fig. 6B, Supplementary Fig. 4 J). The number of OCN-positive osteoblasts and the bone mineralization rate on the trabecular bone surface were reduced in the DO rats treated with either PBS or GMNP_E_-EV-mimic NC + sh-NC as compared to normal rats. However, GMNP_E_-EV-miR-150-5p mimic resulted in elevated number of OCN-positive osteoblasts and bone mineralization rate. Further silencing of β-catenin led to elevations in OCN-positive osteoblasts and bone mineralization rate (Fig. 6C, D).

As illustrated in Fig. 6E, ELISA data exhibited increased CTX-I and TRAP5b serum levels in the DO rats treated with either PBS or GMNP_E_-EV-mimic NC + sh-NC as compared to normal rats, but reduced CTX-I and TRAP5b serum levels in the DO rats treated with GMNP_E_-EV-miR-150-5p mimic. Loss of β-catenin further caused a decrease in the CTX-I and TRAP5b levels.

Furthermore, micro-CT analysis results suggested a decline in the Tb.Th, BV/TV, and Tb.N and an increase in Tb.Sp in the DO rats treated with PBS or GMNP_E_-EV-mimic NC + sh-NC. In the DO rats treated with GMNP_E_-EV-miR-150-5p mimic + sh-NC, Tb.Th, BV/TV, and Tb.N were increased but Tb.Sp was decreased. However, further silencing of β-catenin led to opposite results (Fig. 6F).

In summary, GMNP_E_-EV-miR-150-5p can accelerate osteogenesis of DO rats through the MMP14/Wnt/β-catenin axis.

## Discussion

The data collected from this study supported the pro-proliferative and pro-mature effect of miR-150-5p in BMSC-EV-loaded GMNPs on the osteoblasts via activation of MMP14-mediated Wnt/β-catenin pathway.

We illustrated decreased miR-150-5p in the bone tissues of DO rats while its re-expression could enhance the osteogenesis in DO rats. In line with these findings, a recent study has demonstrated decreased miR-150-3p expression in osteoporosis (Qiu et al. 2021). Notably, our findings displayed that miR-150-5p could elevate the expression of osteogenesis-related genes (RUNX2, BSP, OPN, and OCN) (Wang et al. 2017; Tang et al. 2020), and increased the proportion of OCN-positive osteoblasts and bone mineralization rate, contributing to its pro-mature effect. Meanwhile, it has been confirmed that forced expression of miR-150 in mouse osteoblast cell line MC3T3-E1 leads to increased expression of OC, ALP, type I collagen, and OPN, leading to promotion of bone formation (Dong et al. 2015). These lines of evidence support the promoting property of miR-150-5p in the osteogenesis in DO.

The current study further revealed that miR-150-5p enriched in BMSC-EVs which can carry miR-150-5p to promote the proliferative and mature functions of osteoblasts. Osteoblast maturation can be tested with ALP and markers of osteoblast differentiation (Im et al. 2004; Crous and Abrahamse 2021). A previous study has elaborated the pro-proliferative and pro-differentiation roles of BMSC-EVs in the osteoblasts (Qin et al. 2016). Exosomal miR-150-3p can further strengthen the role of BMSC-derived exosomes in augmenting the proliferating and differentiating potentials of osteoblasts (Qiu et al. 2021), which is consistent with our findings. Considering that, we constructed GMNP_E_ loaded with EV-miR-150-5p and determined the optimal structure of GMNP_E_ to capture EVs. Subsequent results of this study further demonstrated that GMNP_E_ promoted the enrichment of EVs in the bone tissues of DO rats whereby enhancing the proliferative and differentiating properties of osteoblasts. In partial agreement with our results, hydroxyapatite-coated magnetite (Fe_3_O_4_) nanoparticles have been demonstrated to enhance the proliferation and differentiation of osteoblasts by increasing secretion of ALP, collagen, and calcium deposition, thus preventing osteoporosis (Tran et al. 2012; Tran and Webster 2013). These findings were further substantiated in the DO rat models, supporting the enhanced osteoblast functions induced by GMNP_E_-EV-miR-150-5p.

Mechanistic investigations indicated that loss of MMP14, a target gene of miR-150-5p, conferred the pro-proliferative and pro-mature effects of miR-150-5p on osteoblasts. Consistently, miR-150-5p is able to target MMP14 by binding to its 3′-UTR in fibroblast-like synoviocytes in the context of rheumatoid arthritis, thus causing downregulation of its expression (Chen et al. 2018). Aberrant elevation of MMPs can cause a battery of physiological conditions, associated with imbalanced bone remodeling, leading to bone osteolysis or bone formation (Paiva and Granjeiro 2017). Further investigation explained that inhibition of MMP14 activated the Wnt/β-catenin pathway, thus accelerating osteoblast proliferation and maturation. Wnt pathway has been reported to act as an inducer of osteogenic differentiation of MSCs (Yuan et al. 2016). In addition, osteogenesis of human BMSCs can be stimulated following activation of Wnt/β-catenin pathway (Xu et al. 2021). Recent study has proposed that Wnt/β-catenin pathway activation accelerates osteoblast activity, corresponding to increased expression of osteogenesis-related genes RUNX2 and OCN in MC3T3-E1 cells or rat primary osteoblasts (Wang et al. 2020, 2017). Meanwhile, activating the Wnt/β-catenin pathway delays osteoporosis in ovariectomized rats (Liu and Guo 2020). Thus, disruption of the MMP14-mediated blockade of Wnt/β-catenin pathway may contribute to therapeutic potentials in promoting osteogenesis and inhibiting the DO progression.

**Fig. 7:**
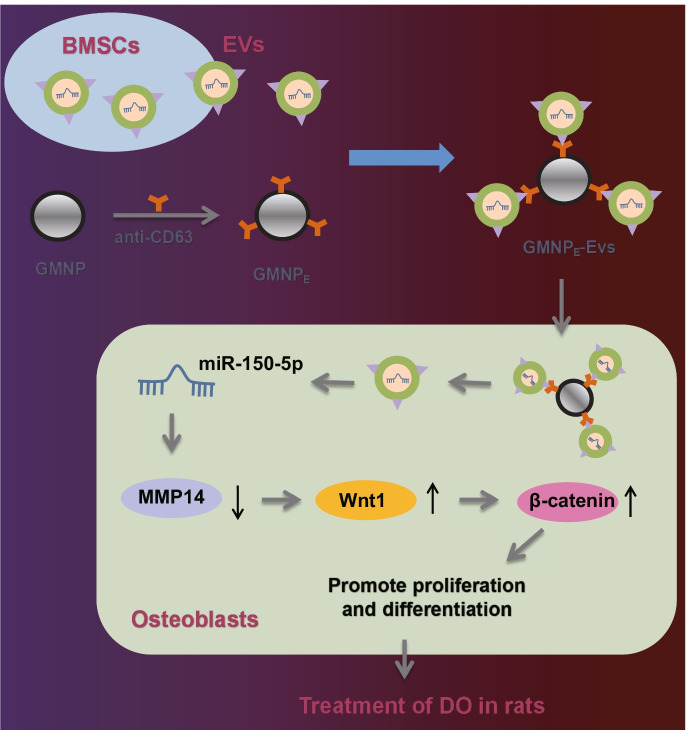
Schematic diagram of the mechanism by which BMSC-EV-loaded GMNPs affect the progression of DO. GMNPs loaded with BMSC-EVs can transfer miR-150-5p to osteoblasts where miR-150-5p binds to MMP14 and results in decreased MMP14 expression, leading to activation of the Wnt/β-catenin pathway. By this mechanism, proliferation and maturation of osteoblasts are enhanced and the DO progression is delayed. The black upward and downward arrows in the figure represent the upregulation and downregulation of gene expression, respectively

Overall, our study indicates that miR-150-5p delivered by BMSC-EV-loaded GMNPs can enhance the osteogenesis through modulating the MMP14-Wnt/β-catenin axis, thus attenuating DO (Fig. 7). Thus, BMSC-EV-mediated transfer of miR-150-5p may act as a potentially effective strategy for diagnosing and monitoring the progression of DO. However, miR-150-5p exerts its therapeutic effect on DO by inhibiting the inflammatory response in DO rats and its specific molecular mechanism require further exploration in future studies. In addition, Wnt/β-catenin regulation is mainly induced by the two Wnt pathway antagonists: SOST and DKK1 (Rossini et al. 2013). Therefore, in addition to targeting MMP14 to activate Wnt/β-catenin, miR-150-5p may also promote osteoblast proliferation and maturation by targeting SOST and DKK1 to activate Wnt/β-catenin, thus alleviating DO. Further investigation is required for validation of this speculation.

## Supplementary Information

Below is the link to the electronic supplementary material.

Supplementary file1 (DOCX 2.98 MB)

Supplementary file2 (DOCX 125 KB)

## Data Availability

The data that supports the findings of this study are available in the manuscript and supplementary materials.

## References

[CR1] An Y, Zhang H, Wang C, Jiao F, Xu H, Wang X (2019). Activation of ROS/MAPKs/NF-kappaB/NLRP3 and inhibition of efferocytosis in osteoclast-mediated diabetic osteoporosis. FASEB J.

[CR2] Cao Y, Qiu Y, Liu MX, Hu Y, Chen FW (2021). MiR-29c-3p reduces bone loss in rats with diabetic osteoporosis via targeted regulation of Dvl2 expression. Eur Rev Med Pharmacol Sci..

[CR3] Chen Z, Wang H, Xia Y, Yan F, Lu Y (2018). Therapeutic potential of mesenchymal cell-derived miRNA-150-5p-expressing exosomes in rheumatoid arthritis mediated by the modulation of MMP14 and VEGF. J Immunol.

[CR4] Cortet B, Lucas S, Legroux-Gerot I, Penel G, Chauveau C, Paccou J (2019). Bone disorders associated with diabetes mellitus and its treatments. Joint Bone Spine.

[CR5] Crous A, Abrahamse H (2021). The signalling effects of photobiomodulation on osteoblast proliferation, maturation and differentiation: a review. Stem Cell Rev Rep.

[CR6] Delgado-Calle J, Hancock B, Likine EF, Sato AY, McAndrews K, Sanudo C (2018). MMP14 is a novel target of PTH signaling in osteocytes that controls resorption by regulating soluble RANKL production. FASEB J.

[CR7] Dominici M, Le Blanc K, Mueller I, Slaper-Cortenbach I, Marini F, Krause D (2006). Minimal criteria for defining multipotent mesenchymal stromal cells. The International Society for Cellular Therapy position statement. Cytotherapy.

[CR8] Dong CL, Liu HZ, Zhang ZC, Zhao HL, Zhao H, Huang Y (2015). The influence of microRNA-150 in osteoblast matrix mineralization. J Cell Biochem.

[CR9] Duan S, Wang F, Cao J, Wang C (2020). Exosomes derived from microRNA-146a-5p-enriched bone marrow mesenchymal stem cells alleviate intracerebral hemorrhage by inhibiting neuronal apoptosis and microglial M1 polarization. Drug Des Devel Ther.

[CR10] Ge J, Zhou Q, Niu J, Wang Y, Yan Q, Wu C (2019). Melatonin protects intervertebral disc from degeneration by improving cell survival and function via activation of the ERK1/2 signaling pathway. Oxid Med Cell Longev.

[CR11] Guan H, Peng R, Mao L, Fang F, Xu B, Chen M (2020). Injured tubular epithelial cells activate fibroblasts to promote kidney fibrosis through miR-150-containing exosomes. Exp Cell Res.

[CR12] Huang Y, Zhang X, Zhan J, Yan Z, Chen D, Xue X (2021). Bone marrow mesenchymal stem cell-derived exosomal miR-206 promotes osteoblast proliferation and differentiation in osteoarthritis by reducing Elf3. J Cell Mol Med.

[CR13] Im GI, Qureshi SA, Kenney J, Rubash HE, Shanbhag AS (2004). Osteoblast proliferation and maturation by bisphosphonates. Biomaterials.

[CR14] Jauregui R, Srinivasan S, Vojtech LN, Gammill HS, Chiu DT, Hladik F (2018). Temperature-responsive magnetic nanoparticles for enabling affinity separation of extracellular vesicles. ACS Appl Mater Interfaces.

[CR15] Kooijmans SAA, Stremersch S, Braeckmans K, de Smedt SC, Hendrix A, Wood MJA (2013). Electroporation-induced siRNA precipitation obscures the efficiency of siRNA loading into extracellular vesicles. J Control Release.

[CR16] Li S, Zhou H, Hu C, Yang J, Ye J, Zhou Y (2021). Total flavonoids of rhizoma drynariae promotes differentiation of osteoblasts and growth of bone graft in induced membrane partly by activating Wnt/beta-catenin signaling pathway. Front Pharmacol.

[CR17] Li T, Xie J, Shen C, Cheng D, Shi Y, Wu Z (2014). miR-150-5p inhibits hepatoma cell migration and invasion by targeting MMP14. PLoS ONE.

[CR18] Liao Z, Luo R, Li G, Song Y, Zhan S, Zhao K (2019). Exosomes from mesenchymal stem cells modulate endoplasmic reticulum stress to protect against nucleus pulposus cell death and ameliorate intervertebral disc degeneration in vivo. Theranostics.

[CR19] Liu B, Wang B, Zhang X, Lock R, Nash T, Vunjak-Novakovic G. Cell type-specific microRNA therapies for myocardial infarction. Sci Transl Med. 2021;13: 10.1126/scitranslmed.abd0914 10.1126/scitranslmed.abd0914 PMC8848299 33568517

[CR20] Liu S, Chen X, Bao L, Liu T, Yuan P, Yang X (2020). Treatment of infarcted heart tissue via the capture and local delivery of circulating exosomes through antibody-conjugated magnetic nanoparticles. Nat Biomed Eng.

[CR21] Liu TJ, Guo JL (2020). Overexpression of microRNA-141 inhibits osteoporosis in the jawbones of ovariectomized rats by regulating the Wnt/beta-catenin pathway. Arch Oral Biol.

[CR22] Liu Z, Sun J, Li C, Xu L, Liu J (2021). MKL1 regulates hepatocellular carcinoma cell proliferation, migration and apoptosis via the COMPASS complex and NF-kappaB signaling. BMC Cancer.

[CR23] Livak KJ, Schmittgen TD (2001). Analysis of relative gene expression data using real-time quantitative PCR and the 2(-delta delta C(T)) method. Methods.

[CR24] Lu H, Hu L, Yu L, Wang X, Urvalek AM, Li T (2014). KLF8 and FAK cooperatively enrich the active MMP14 on the cell surface required for the metastatic progression of breast cancer. Oncogene.

[CR25] Mohsin S, Baniyas MM, AlDarmaki RS, Tekes K, Kalasz H, Adeghate EA (2019). An update on therapies for the treatment of diabetes-induced osteoporosis. Expert Opin Biol Ther.

[CR26] Mori MA, Ludwig RG, Garcia-Martin R, Brandao BB, Kahn CR (2019). Extracellular miRNAs: from biomarkers to mediators of physiology and disease. Cell Metab.

[CR27] Oliveira-Rodriguez M, Serrano-Pertierra E, Garcia AC, Lopez-Martin S, Yanez-Mo M, Cernuda-Morollon E (2017). Point-of-care detection of extracellular vesicles: sensitivity optimization and multiple-target detection. Biosens Bioelectron.

[CR28] Paiva KBS, Granjeiro JM (2017). Matrix metalloproteinases in bone resorption, remodeling, and repair. Prog Mol Biol Transl Sci.

[CR29] Pang X, Zhong Z, Jiang F, Yang J, Nie H (2022). Juglans regia L extract promotes osteogenesis of human bone marrow mesenchymal stem cells through BMP2/Smad/Runx2 and Wnt/beta-catenin pathways. J Orthop Surg Res.

[CR30] Qin Y, Wang L, Gao Z, Chen G, Zhang C (2016). Bone marrow stromal/stem cell-derived extracellular vesicles regulate osteoblast activity and differentiation in vitro and promote bone regeneration in vivo. Sci Rep.

[CR31] Qiu M, Zhai S, Fu Q, Liu D (2021). Bone marrow mesenchymal stem cells-derived exosomal microRNA-150-3p promotes osteoblast proliferation and differentiation in osteoporosis. Hum Gene Ther.

[CR32] Rani S, Ryan AE, Griffin MD, Ritter T (2015). Mesenchymal stem cell-derived extracellular vesicles: toward cell-free therapeutic applications. Mol Ther.

[CR33] Rehling T, Bjorkman AD, Andersen MB, Ekholm O, Molsted S (2019). Diabetes is associated with musculoskeletal pain, osteoarthritis, osteoporosis, and rheumatoid arthritis. J Diabetes Res.

[CR34] Rossini M, Gatti D, Adami S (2013). Involvement of WNT/beta-catenin signaling in the treatment of osteoporosis. Calcif Tissue Int.

[CR35] Schacter GI, Leslie WD (2017). Diabetes and bone disease. Endocrinol Metab Clin North Am.

[CR36] Sharma AR, Nam JS (2019). Kaempferol stimulates WNT/beta-catenin signaling pathway to induce differentiation of osteoblasts. J Nutr Biochem.

[CR37] Tang CY, Chen W, Luo Y, Wu J, Zhang Y, McVicar A (2020). Runx1 up-regulates chondrocyte to osteoblast lineage commitment and promotes bone formation by enhancing both chondrogenesis and osteogenesis. Biochem J.

[CR38] Tran N, Hall D, Webster TJ (2012). Mechanisms of enhanced osteoblast gene expression in the presence of hydroxyapatite coated iron oxide magnetic nanoparticles. Nanotechnology.

[CR39] Tran N, Webster TJ (2013). Understanding magnetic nanoparticle osteoblast receptor-mediated endocytosis using experiments and modeling. Nanotechnology.

[CR40] Wang-Fischer Y, Garyantes T (2018). Improving the reliability and utility of streptozotocin-induced rat diabetic model. J Diabetes Res.

[CR41] Wang Q, Shi D, Geng Y, Huang Q, Xiang L (2020). Baicalin augments the differentiation of osteoblasts via enhancement of microRNA-217. Mol Cell Biochem.

[CR42] Wang Y, Zhang X, Shao J, Liu H, Liu X, Luo E (2017). Adiponectin regulates BMSC osteogenic differentiation and osteogenesis through the Wnt/beta-catenin pathway. Sci Rep.

[CR43] Wu K, Su D, Liu J, Saha R, Wang JP (2019). Magnetic nanoparticles in nanomedicine: a review of recent advances. Nanotechnology.

[CR44] Xu Y, Jiang Y, Jia B, Wang Y, Li T (2021). Icariin stimulates osteogenesis and suppresses adipogenesis of human bone mesenchymal stem cells via miR-23a-mediated activation of the Wnt/beta-catenin signaling pathway. Phytomedicine.

[CR45] Yang C, Wang C, Zhou J, Liang Q, He F, Li F (2020). Fibronectin 1 activates WNT/beta-catenin signaling to induce osteogenic differentiation via integrin beta1 interaction. Lab Invest.

[CR46] Yuan Z, Li Q, Luo S, Liu Z, Luo D, Zhang B (2016). PPARgamma and Wnt signaling in adipogenic and osteogenic differentiation of mesenchymal stem cells. Curr Stem Cell Res Ther.

[CR47] Zhao CY, Chen JT, Yang DH, Zhong ZM, Bai L (2010). Effects of extracts of oxyntic mucosa in rat on the biological activity of osteoblasts. Osteoporos Int.

[CR48] Zhou J, Li X, Wu X, Zhang T, Zhu Q, Wang X (2018). Exosomes released from tumor-associated macrophages transfer miRNAs that induce a Treg/Th17 cell imbalance in epithelial ovarian cancer. Cancer Immunol Res.

[CR49] Zhu J, Liu B, Wang Z, Wang D, Ni H, Zhang L (2019). Exosomes from nicotine-stimulated macrophages accelerate atherosclerosis through miR-21-3p/PTEN-mediated VSMC migration and proliferation. Theranostics.

[CR50] Zuo R, Kong L, Wang M, Wang W, Xu J, Chai Y (2019). Exosomes derived from human CD34(+) stem cells transfected with miR-26a prevent glucocorticoid-induced osteonecrosis of the femoral head by promoting angiogenesis and osteogenesis. Stem Cell Res Ther.

