# Supplementary material for: Extracellular vesicles derived from bone marrow mesenchymal stem cells loaded on magnetic nanoparticles delay the progression of diabetic osteoporosis via delivery of miR-150-5p
Source: Cell Biol Toxicol. 2022 Sep 16;39(4):1257–74. doi: 10.1007/s10565-022-09744-y (PMC10425527; doi:10.1007/s10565-022-09744-y)
Supplement: Supplementary file 1 — Supplementary file1 (DOCX 2.98 MB) [file 10565_2022_9744_MOESM1_ESM.docx]

**
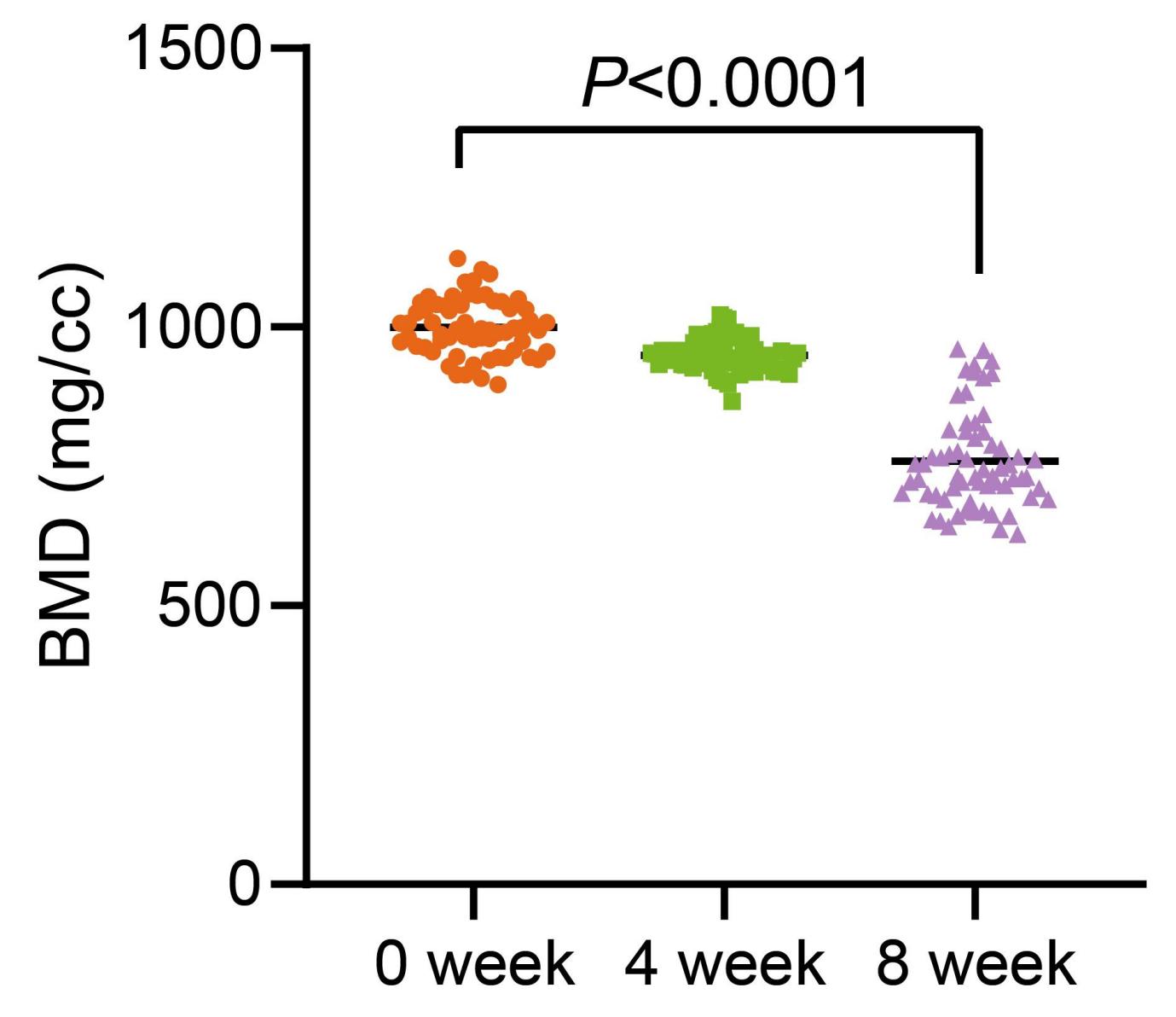
**

**Supplementary Figure 1** Detection of the BMD of 64 rats used for model establishment every 4 weeks. * *p <* 0.05.

**
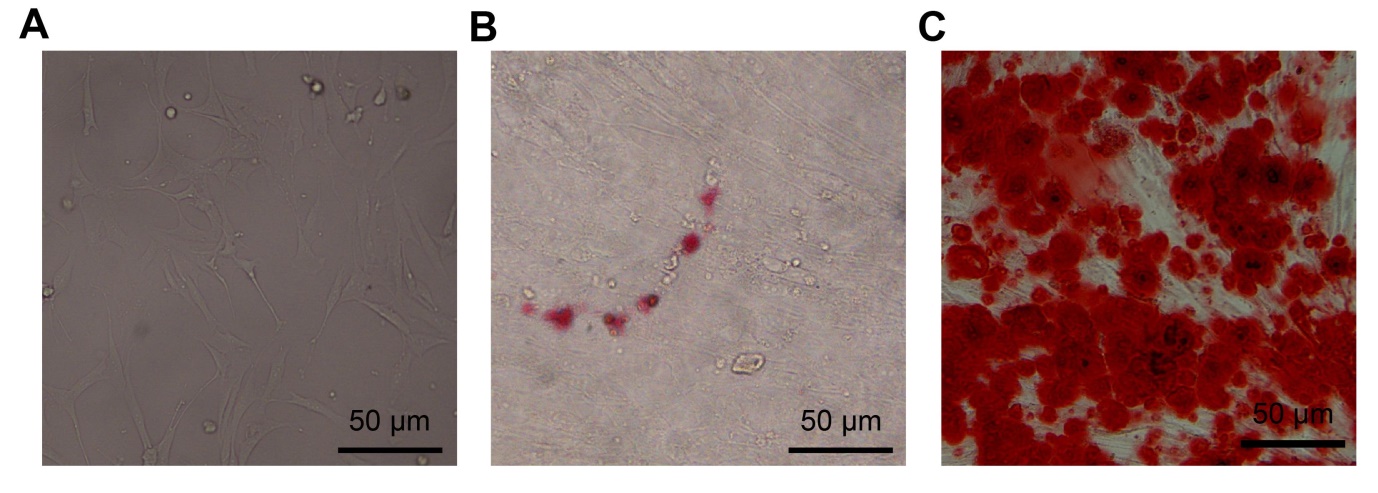
**

**Supplementary Figure 2** BMSC identification. A, Microscopic images showing the morphology of BMSCs after 72 h of culture. B, Oil red O staining of BMSCs after 3 weeks of adipogenic induction. C, Alizarin red S staining of BMSCs after 3 weeks of adipogenic induction. The cell experiment was repeated 3 times independently.

**
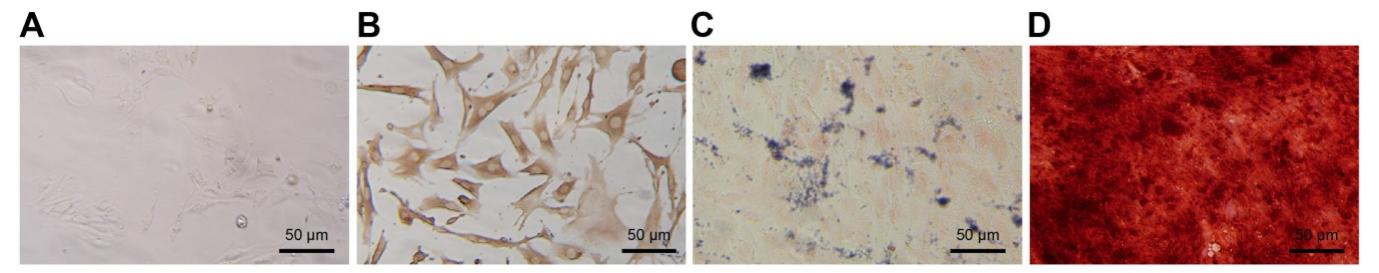
**

**Supplementary Figure 3** Osteoblast identification. A, Microscopic images showing osteoblast morphology. B, Immunohistochemistry analysis of type I collagen protein in the osteoblasts. C, ALP staining images of the osteoblasts; D, Alizarin red S staining images of the osteoblasts. The cell experiment was repeated 3 times independently.

**
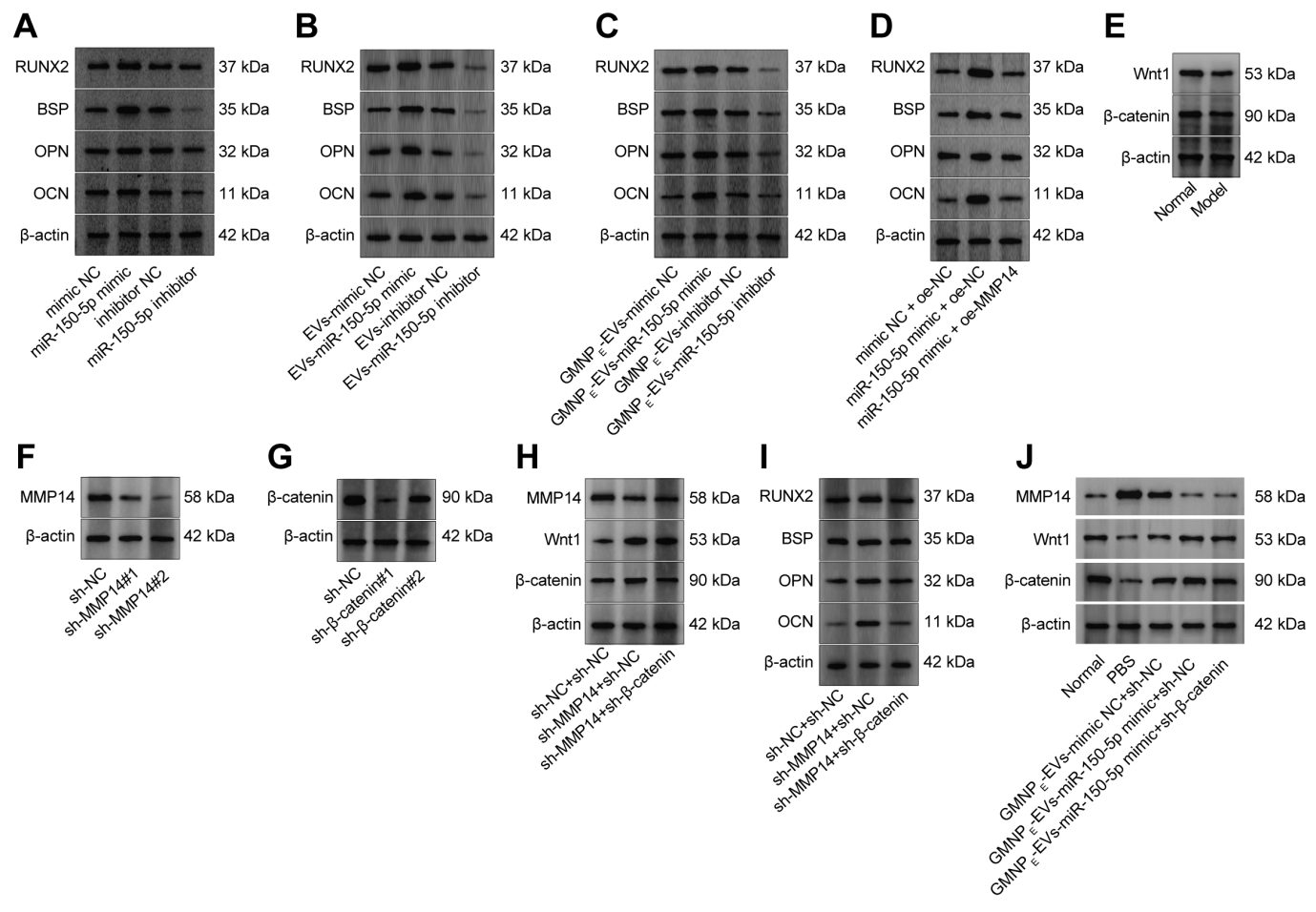
**

**Supplementary Figure 4** Representative immunoblots for quantification of the band intensities of Figure 1E (A), Figure 2K (B), Figure 3E (C), Figure 4K (D), Figure 5D (E), Figure 5E (F), Figure 5F (G), Figure 5G (H), Figure 5L (I) and Figure 6B (J). The cell experiment was repeated 3 times independently.

**
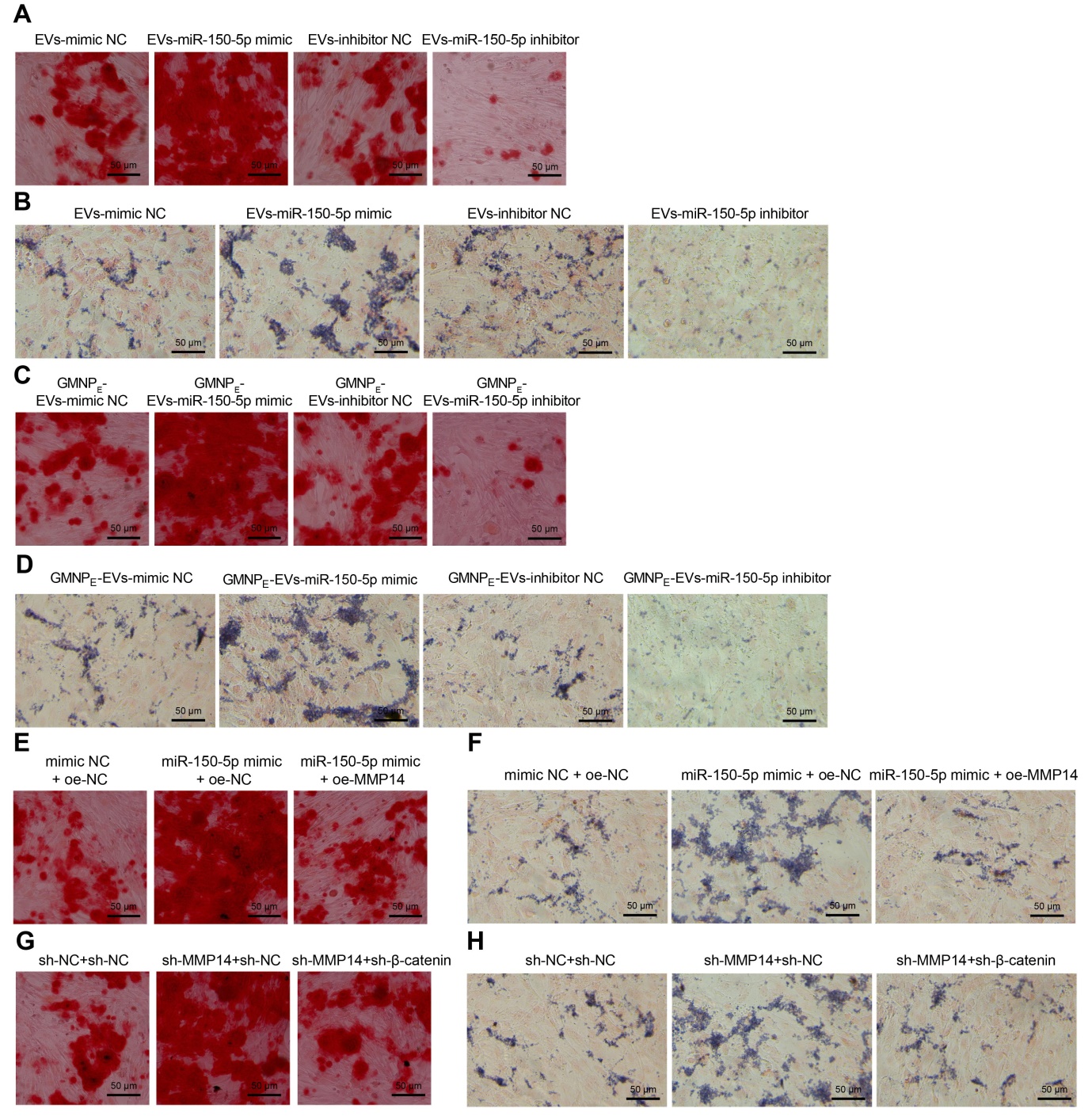
**

**Supplementary Figure 5** Representative alizarin red S staining images of Figure 2H (A), Figure 3B (C), Figure 4H (E), and Figure 5I (G), as well as the representative ALP staining images of Figure 2I (B), Figure 3C (D), Figure 4I (F) and Figure 5J (H).

**
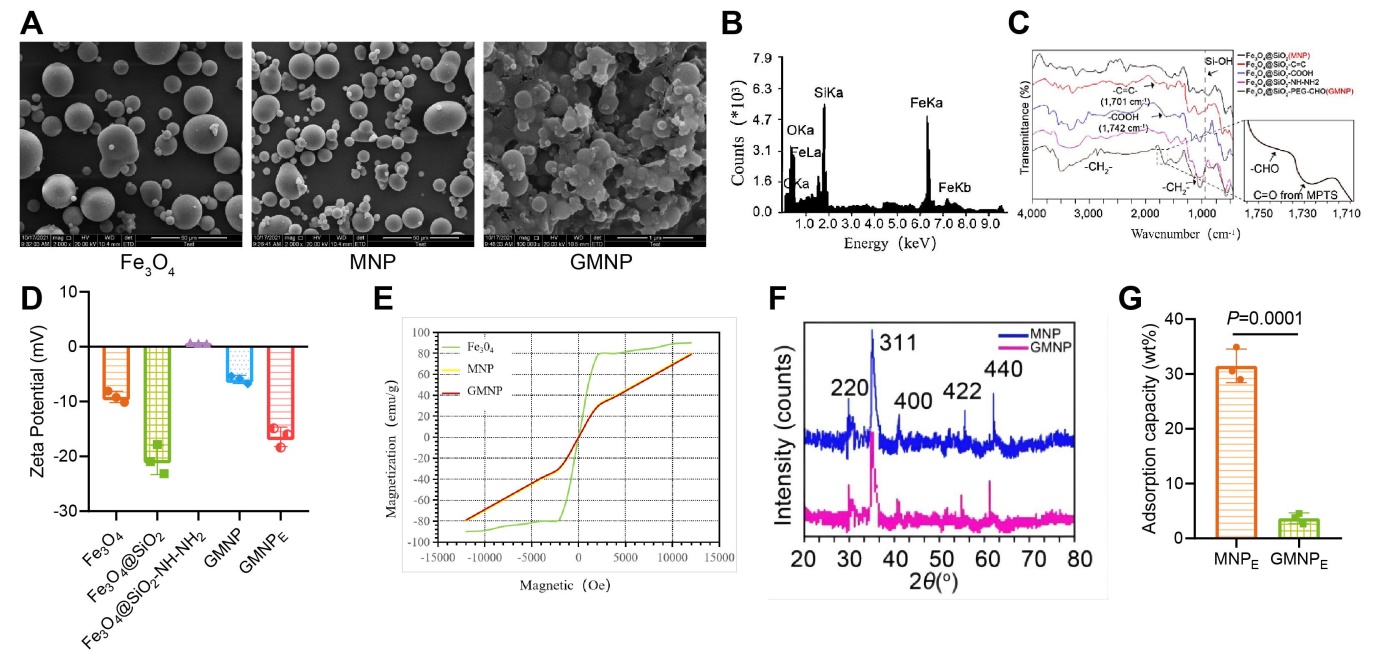
**

**Supplementary Figure 6** Synthesis and identification of GMNPs. A, SEM images of size of Fe3O4, MNPs, and GMNPs. B, Energy dispersive spectrum analysis of GMNPs. C, Infrared spectroscopy analysis of the chemical composition of GMNPs in each synthesis step. D, Zeta potential of GMNP_E_ before and after modification. E, Hysteresis loop of GMNPs before and after modification. F, X-ray diffraction pattern of GMNPs before and after modification. G, Fluorescence spectrometry measurement of HSA adsorption amount in the GMNP_E_ and MNP_E_. * *p* < 0.05.

**
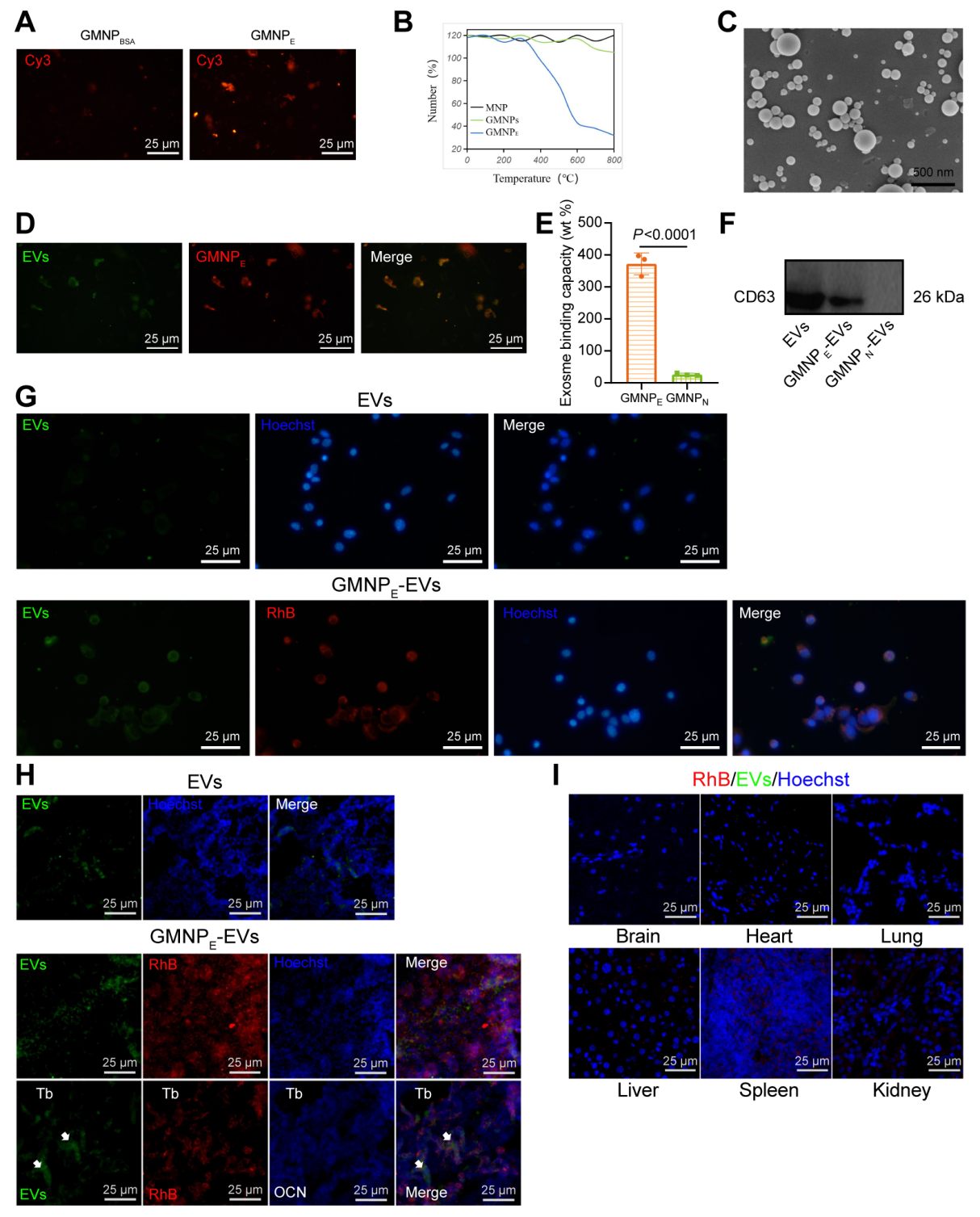
**

**Supplementary Figure 7** GMNP_E_ contributes to the enrichment of EVs in the bone tissues of DO rats. A, Confocal microscopic images of anti-CD63 antibody on the surface of GMNP_E_. B, TGA was used to detect the antibody content of GMNP_E_. C, Structure of GMNP_E_-EVs observed under a TEM. D, Confocal microscopic images of GMNP_E_-EVs, wherein magnetic nanoparticles are visualized in red and EVs in green. E, Binding ability of GMNP_E_ and GMNP_N_ to EVs. F, Immunoblotting of EV marker protein CD63 in GMNP_E_-EVs, GMNP_N_-EVs and EVs samples. G, Confocal microscopic images of the localization of GMNP_E_-EVs and EVs in osteoblasts. H, Confocal microscopic images of the localization of GMNP_E_-EVs and EVs in the femoral tissues of DO rats. Tb indicates trabecular bone, and white arrows indicate co-localized cells. I, GMNP_E_ (red)-EV (green) signal in cells of the brain, heart, lung, liver, spleen and kidney tissues detected by immunofluorescence staining. * *p* < 0.05. The cell experiment was repeated 3 times independently.
