# Supplementary material for: Extracellular vesicles derived from bone marrow mesenchymal stem cells loaded on magnetic nanoparticles delay the progression of diabetic osteoporosis via delivery of miR-150-5p
Source: Cell Biol Toxicol. 2022 Sep 16;39(4):1257–74. doi: 10.1007/s10565-022-09744-y (PMC10425527; doi:10.1007/s10565-022-09744-y)
Supplement: Supplementary file 2 — Supplementary file2 (DOCX 125 KB) [file 10565_2022_9744_MOESM2_ESM.docx]

**Supplementary Table 1** shRNA sequences

| shRNA | Sequence (5’-3’) |
| --- | --- |
| sh-MMP14#1 (rat) | GCAAAGGTTCTATGGTTTACA |
| sh-MMP14#2 (rat) | GCTGTGGTGTTCCAGATAAGT |
| sh-MMP14#3 (rat)） | GCTTCTACCACAAGGACTTTG |
| sh-β-catenin#1 (rat) | GCTTGTTGGCCATCTTTAAAT |
| sh-β-catenin#2 (rat)） | GCTGCATAATCTCCTGCTACA |
| sh-β-catenin#3 (rat) | GGACTACAAGAAACGGCTTTC |

**Supplementary Table 2** Primer sequences for reverse transcription quantitative polymerase chain reaction

| Gene | Sequence (5’-3’) |
| --- | --- |
| miR-150-5p (rat) | Forward: TCTCCCAACCCTTGTACCA |
|  | Reverse: universal reverse primer |
| MMP14 (rat) | Forward: GAGTATGGGAGAGTGCCACG |
|  | Reverse: AAAGTGGGTATCCCCTCCGA |
| β-catenin (rat) | Forward: ATCATTCTGGCCAGTGGTGG |
|  | Reverse: GACAGCACCTTCAGCACTCT |
| RUNX2 (rat) | Forward: CGCCTCACAAACAACCACAG |
|  | Reverse: AATGACTCGGTTGGTCTCGG |
| BSP (rat) | Forward: CAGGACTGCCGAAGGAAGAAA |
|  | Reverse: TCGAGAAAGCACTCGCCATC |
| U6 | Forward: CTCGCTTCGGCAGCACA |
|  | Reverse: universal reverse primer |
| GAPDH (rat) | Forward: AGACAGCCGCATCTTCTTGT |
|  | Reverse: TACGGCCAAATCCGTTCACA |

**Supplementary Table 3** Components of nano-particles

| Abbreviation | Component |
| --- | --- |
| MNP | Fe_3_O_4_@SiO_2_ |
| MNP_E_ | Fe_3_O_4_@SiO_2_，anti-CD63 |
| GMNP | Fe_3_O_4_@SiO_2_-PEG-CHO |
| GMNP_E_ | Fe_3_O_4_@SiO_2_-PEG-CHO, anti-CD63 |
| GMNP_N_ | Fe_3_O_4_@SiO_2_-PEG-CHO, IgG isotype control |
| GMNP_BSA_ | Fe_3_O_4_@SiO_2_-PEG-CHO, BSA |
